# Bioactive Compounds, Antioxidant Activity, and Antinutritional Content of Legumes: A Comparison between Four *Phaseolus* Species

**DOI:** 10.3390/molecules25153528

**Published:** 2020-08-01

**Authors:** Montserrat Alcázar-Valle, Eugenia Lugo-Cervantes, Luis Mojica, Norma Morales-Hernández, Heidy Reyes-Ramírez, Jhony Navat Enríquez-Vara, Soledad García-Morales

**Affiliations:** 1 Centro de Investigación y Asistencia en Tecnología y Diseño del Estado de Jalisco, A.C., 45019 Zapopan, Mexico; ealcazar@ciatej.mx (M.A.-V.); elugo@ciatej.mx (E.L.-C.); lmojica@ciatej.mx (L.M.); nmorales@ciatej.mx (N.M.-H.); heidyr798@gmail.com (H.R.-R.); 2 CONACYT-Centro de Investigación y Asistencia en Tecnología y Diseño del Estado de Jalisco A.C., 45019 Zapopan, Mexico; jenriquez@ciatej.mx

**Keywords:** anthocyanins, bioactive compounds, essential amino acid, flavonoids, nutritional content, *Phaseolus* spp., phenolic compounds, proximate composition

## Abstract

Beans (*Phaseolus* spp.) are one of the most important legumes for their nutritional value and health benefits in many world regions. In addition to *Phaseolus vulgaris*, there are four additional species that are cultivated in many regions of the world and are a source of food for human consumption: *P. lunatus*, *P. coccineus*, *P. polyanthus*, and *P. acutifolius.* In this work, phenolic compounds, antioxidant activity, and anti-nutritional compounds of 18 bean accessions, corresponding to four different species of the genus *Phaseolus*, were analyzed. In addition, their physical characteristics, proximate composition, and amino acid content were determined in order to compare their phytochemical composition and nutritional value. The species closest to each other in terms of essential amino acid content were *P. polyanthus* with *P. vulgaris* and *P. lunatus* with *P. coccineus.* Furthermore, there was a strong positive correlation between antioxidant activity and flavonoids, anthocyanins, and lectins with all the accessions collected. Significant differences in the content of phenolic compounds were found among the bean species studied. Therefore, in addition to *P. vulgaris*, other species such as *P. coccineus* and *P. lunatus* have high biological and antioxidant potential that could be beneficial to human health when consumed as nutraceutical foods.

## 1. Introduction

Beans (*Phaseolus* spp.) belong to the legume family (*Fabaceae*), along with peas (*Pisum sativum L.*), soybeans (*Glycine max* (L.) Merril), and lentils (*Lens culinaris* Medik). Beans are the most important legume and are consumed by approximately 300 million people. Moreover, they are considered the second source of protein in East and Southeast Africa and the fourth source in America [1].

There are about 150 known species of beans around the world. In Mexico alone, there are approximately 65 species, 52 of which belong to the *Phaseolus* genus, and 31 species are endemic [2]. The largest number of species are distributed in Western Mexico, with *P. vulgaris* being the most studied species. Thus, different studies have quantified the content of phytochemicals in some varieties of *P. vulgaris*, such as: Total phenols, anthocyanins, tannins, flavonoids, lectins, phytic acid, and oligosaccharides [3,4]. In addition to the protein, carbohydrate, fiber, mineral, and vitamin contents [5].

These investigations agree that *P. vulgaris* varieties have high protein, carbohydrate, fiber, mineral, and vitamin contents. In addition, high concentration of phenolic compounds, such as flavonoids, anthocyanins, and tannins, have biological activity. However, it has been observed that the content of phenolic compounds can vary between bean seeds, with dark seeds having the highest concentration compared to the light-colored varieties [6]. Furthermore, some authors mentioned that polyphenols, flavonoids, and anthocyanins present in different varieties of *P. vulgaris* have antioxidant properties [2,7].

Additionally, some clinical studies have shown beneficial effects from the consumption of different varieties of *P. vulgaris,* such as a reduction in the glycemic index, in cardiovascular diseases, stomach and prostate cancer, in weight control, and in obesity [2]. For example, kaempferol and quercetin are the main flavonoids in *P. vulgaris,* and both compounds could be beneficial to reduce cardiovascular disease; genistein, an isoflavonoid, may inhibit the growth of carcinogenic cells, including breast and prostate cancer, and anthocyanins such as cyanidin 3-glucoside exhibit antioxidant activity [3,8]. Besides, some varieties of *P. vulgaris* contain ferulic acid as their main phenolic acid, which is also characterized by its antioxidant activity [2].

From an antinutritional point of view, low protein digestibility is an important nutritional problem of common beans (*P. vulgaris*), which is commonly attributed to the presence of trypsin inhibitors that decrease protein efficiency, reduce food gain and can be an occupational allergen. Tannins can also reduce food consumption, growth rate and the bioavailability of minerals [9].

Other antinutritional components of beans are lectins that induce the growth of the pancreas, producing ulceration and necrosis in the intestinal epithelium of rats. Phytic acid reduces the bioavailability of essential nutrients such as minerals, proteins, and amino acids. Oligosaccharides, which are metabolized in lower intestine by bacteria that produce methane, hydrogen, and carbon dioxide, can cause flatulence and diarrhea [10].

Nevertheless, to the best of our knowledge, there is little evidence of phenolic compound concentrations and no evidence of the antinutritional composition of other species, such as *P. coccineus*, *P. lunatus,* and *P. polyanthus*. Therefore, due to the social and nutritional importance of these bean species, more attention and studies are needed to identify compounds with biological activity that, by their content of antioxidants, antimutagens, and anticarcinogens, prevent or reduce the risk of chronic degenerative diseases or that may act as cholesterol reducers or may contain compounds that promote glucose tolerance and thus improve the food security and nutrition of people who consume these types of legumes. 

This work aimed to determine the physical characteristics (seed coat color and grain size), proximate composition (moisture, ash, fats, proteins, and total carbohydrates), amino acid content, phenolic compounds (total phenols, flavonoids, anthocyanins, and condensed tannins), antioxidant activity, and antinutritional compounds (phytic acid, oligosaccharides, lectin, and trypsin inhibitory activity) in four bean species collected in the Mayan region of Mexico and compare their chemical composition, nutritional value and antioxidant activity. In order to compare the species *P. vulgars*, *P. coccineus*, *P. lunatus*, and *P. polyanthus* according to their chemical, nutritional and nutraceutical composition; and thus promote the consumption of the less exploited species of the genus *Phaceolus*.

## 2. Results

### 2.1. Physical Characteristics of Phaseolus Species

In this work, eighteen native bean accessions from the Mayan region in Chiapas, Mexico, were studied. Thirteen are *P. vulgaris* species (CH-01, CH-02, CH-03, CH-04, CH-05, CH-08, CH-09, CH-10, CH-12, CH-13, CH-14, CH-15, CH-18), two are *P. coccineus* species (CH-07, CH-16), two are *P. polyanthus* species (CH-06, CH-11), and one is a *P. lunatus* species (CH-17).

The bean accessions were grouped into red, black, white, purple, pink, yellow, and brown tones, and the accession that presented the greatest luminosity (69.43 ± 0.81%) was CH-08 (*P. vulgaris*). Moreover, the luminosity (*L) range from the accessions collected ranged from 69.43 ± 0.81% to 15.30 ± 0.38%; the chromaticity (c) ranged from 28.33 ± 0.22% to 0.11 ± 0.06%, and the hue (h) ranged from 1.48 ± 0.01% to −1.48 ± 0.08 (Table 1). Luminosity (*L) is the radiant intensity per unit area and is associated with the color black to white. Chromaticity (c) is an index similar to saturation and indicates the degree of deviation from grey to pure chromatic color. The hue (h) angle is measured from pure red to orange, yellow, green, etc. [11].

*P. vulgaris* accessions exhibited the most diverse range of color from black, red, brown and white tones, as 13 accessions out of 18 correspond to this species; the accessions with purple tones were both *P. coccineus* samples (CH-07 and CH-16); *P. polyanthus* accessions presented yellow (CH-06) and red (CH-11) tones; and *P. lunatus* accessions presented a pink tone (CH-17).

Furthermore, of the accessions collected, 22% were small, 33% were medium, and 45% were large. The *P. polyanthus* and *P. coccineus* species were the widest, and *P. lunatus* showed the greatest thickness, while *P. coccineus* was the longest. However, *P. vulgaris* accessions were distinguished as the most heterogeneous species in terms of size, since a higher number of collected accessions of this species were found in the Mayan region of Mexico (Table 1). 

### 2.2. Proximate Composition of Bean Seeds

**Figure 1:**
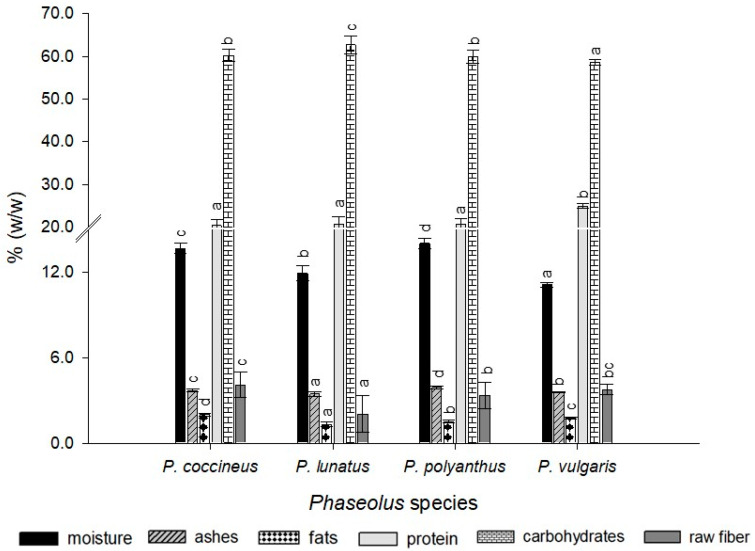
Proximate composition of bean varieties by *Phaseolus* species. Mean values ± standard deviation (SD). Different letters indicate statistically significant differences according to Tukey’s test (*p* < 0.05).

Carbohydrates were the predominant element in bean seeds of all accessions (Figure 1). Furthermore, according to analysis of variance, there was a significant difference between the bean species in terms of their percent moisture, ash, fats, and protein content (*p*-value < 0.05). Moreover, *P. coccineus* accessions had a higher percent by weight of raw fiber and fat, but a lower amount of protein than *P. vulgaris;* whereas, *P. lunatus* had a high percent of carbohydrates and a low amount of ash, fats and raw fiber. However, the accessions from *P. polyanthus* presented a greater composition of moisture and ash. Finally, the *P. vulgaris* accessions presented a higher percent of protein and a lower amount of moisture and carbohydrates (Figure 1).

### 2.3. Essential Amino Acids

**Figure 2:**
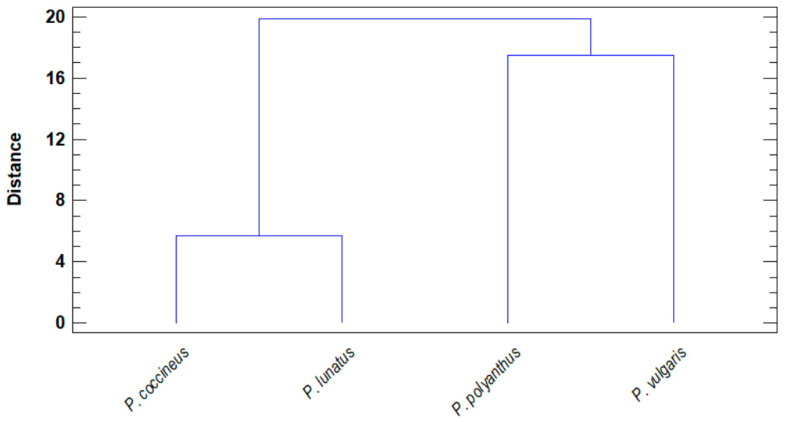
Dendrogram of the closest *Phaseolus* species based on free essential amino acids.

One of the main traits among the bean species studied is the content of essential amino acids. It was observed that there is a closeness between certain species, which in turn show certain distance from the rest of the other species, clustering in two main clades. The closest species to each other in terms of essential amino acids were *P. coccineus* with *P. lunatus* and *P. polyanthus* with *P. vulgaris* (Figure 2). The latter two species showed a higher concentration of essential amino acids. Arginine was the major amino acid, ranging from 661.25 to 115.67 mg/100 g bean flour, and in descending order were tryptophan (108.06–10.34 mg/100 g), histidine (61.7–6.35 mg/100 g), threonine (38.34–8.59 mg/100 g), valine (37.17–8.49 mg/100 g), lysine (28.09–7.99 mg/100 g), leucine (26.92–11.45 mg/100 g), phenylalanine (18.44–4.11 mg/100 g), isoleucine (12.81–2.64 mg/100 g), and methionine (6.98–0.81 mg/100 g). 

Histidine and isoleucine presented a significant difference between *P. coccineus, P. polyanthus,* and *P. vulgaris* (*p*-value < 0.05). *P. polyanthus* was distinguished from the other species by its high content of threonine, valine, and isoleucine. While, *P. vulgaris* had the highest content of histidine, arginine, and tryptophan. In general, *P. coccineus* contained the lowest proportion of amino acids (Table 2).

### 2.4. Phenolic Compounds and Antioxidant Activity

#### 2.4.1. Total Phenols

**Figure 3:**
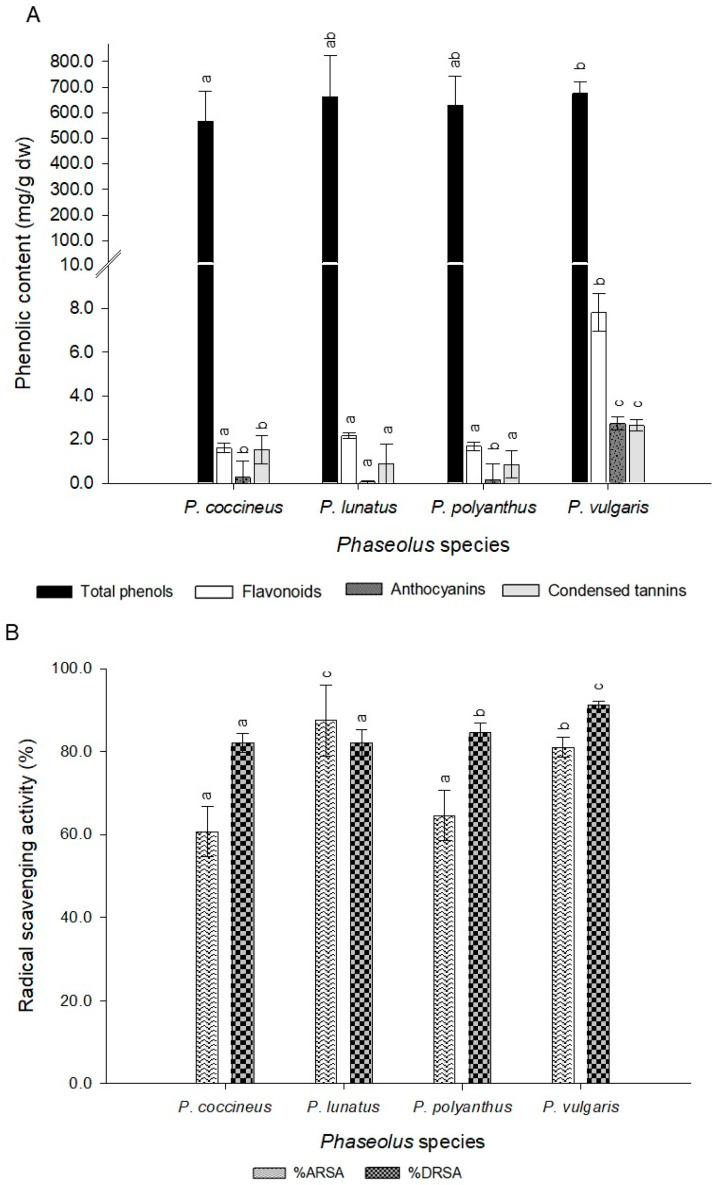
Phenolic compounds (**A**) and antioxidant activity (**B**) of bean varieties by *Phaseolus* species. Mean values ± SD with different letters indicate statistically significant differences according to Tukey’ test (*p* < 0.05). Abbreviations: ARSA = ABTS Radical Scavenging Activity, DRSA = DPPH Radical Scavenging Activity.

The phenolic compound concentrations ranged from 1364.54 ± 0.5 to 114.9 ± 0.7 mg GAE/g dw (Table 3), where sample CH-09 (*P. vulgaris*, red seed hull) presented the highest concentration. The species concentration of total phenols in descending order was *P. vulgaris* > *P. lunatus* > *P. polyanthus* > *P. coccineus*. However, significant differences in total phenol concentration were found between bean species *P. vulgaris* and *P. coccineus* (Figure 3). 

#### 2.4.2. Flavonoids

The flavonoid concentration of the accessions ranged from 19.94 ± 0.47 to 1.09 ± 0.33 mg QE/g dw (Table 3), and CH-09 (*P. vulgaris*, red seed hull) showed the highest concentration. Hence, the concentration of flavonoids in descending order was *P. vulgaris* > *P. lunatus* > *P. coccineus* > *P. polyanthus.* Furthermore, there were significant differences between the bean species and their flavonoid concentrations (*p*-value = 0.007). Thus, the accessions collected from *P. coccineus*, *P. lunatus*, and *P. polyanthus* species showed significant differences with *P. vulgaris* accessions (Figure 3).

#### 2.4.3. Condensed Tannins

The concentration of tannins ranged from 0.53 ± 0.05 to 5.57 ± 0.02 mg CAE/g dw (Table 3). CH-10 (*P. vulgaris,* black) showed the highest concentration of condensed tannins. Moreover, red and black accessions of *P. vulgaris* showed a high concentration of tannins, followed by both accessions of *P. coccineus* (purple) and *P. lunatus* (pink). Both *P. polyanthus* (yellow and red) and *P. vulgaris* (brown and white) accessions had the lowest tannin content. As a result, the species *P. polyanthus* and *P. lunatus* displayed no significant differences, but there were a significant difference (*p* < 0.05) with *P. coccineus* and *P. vulgaris* (Figure 3).

#### 2.4.4. Anthocyanins

The concentration of anthocyanins in the bean seed coat oscillated between not being detecting and 9.42 ± 0.03 mg of cyanidin 3-glucoside per gram of the bean seed coat (Table 3). Similar to tannins, CH-10 (*P. vulgaris,* black) showed the highest concentration of anthocyanins. Moreover, the red and black accessions of *P. vulgaris* species showed a high concentration of anthocyanins, followed by both species of *P. coccineus* (purple) and *P. vulgaris* (brown); both species of *P. polyanthus* (yellow and red), *P. lunatus* (pink), and *P. vulgaris* (white) did not show any anthocyanin content (Table 3).

In addition, when a multiple range analysis of the means in terms of tannins and seed coat color was performed, it was observed that there were significant differences between the black genotype and the white, brown, and red genotypes from all the species collected, with the black seeds presenting the highest tannin contents. Moreover, there is a positive correlation between the anthocyanins and the tannin content (Table 4).

**Figure 4:**
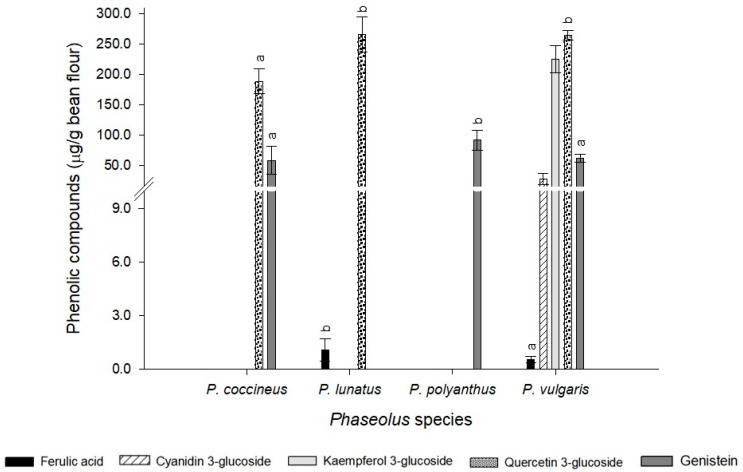
Phenolic compounds analyzed by UHPLC by *Phaseolus* species. Mean values ± SD with different letters indicate statistically significant differences according to Tukey’ test (*p* < 0.05).

In the present study, the content of cyanidin 3-glucoside, genistein, ferulic acid, quercetin 3-glucoside, and kaempferol 3-glucoside was determined. Statistical differences were observed between the analyzed compounds (*p*-value = 0.031) (Figure 4). Cyanidin and kaempferol were only detected in *P. phaseolus*; whereas, quercetin was quantified in three of the four species analyzed (*P. coccineus*, *P. lunatus*, and *P. vulgaris*). Similarly, genistein was found in *P. coccineus*, *P. polyanthus* and *P. vulgaris*. Ferulic acid was observed in *P. lunatus* and P*. vulgaris*. In the species *P. polyanthus* only genistein was identified while in *P. vulgaris* all the phenolic compounds analyzed were detected (Figure 4).

#### 2.4.5. Antioxidant Activity

Accession CH-18 (96.9% ARSA; 98.2% DRSA) showed the highest antioxidant activity in both the ABTS and DPPH assays. However, the accessions with the lowest antioxidant activity were CH-04 from the ABTS assay (56.7% ARSA) and CH-16 from the DPPH assay (78.9% DRSA). However, all bean accessions showed antioxidant activity with both methodologies (Table 3), but no significant differences were observed between the phenolic compounds and the antioxidant activity with the ABTS technique. 

Also, Pearson’s correlation coefficient between antioxidant activity (DPPH test) and bioactive compounds found in all collected accessions was determined (Table 4). A significant correlation coefficient (*p*-value < 0.05) was found between DPPH and flavonoids (0.95), anthocyanins (0.96), or lectin (0.97).

### 2.5. Antinutritional Components

The phytic acid concentration oscillated from 24.09±0.38 to 158.15 ± 0.53 mg of phytic acid per 100 g of bean flour (Table 3). CH-10 (*P. vulgaris,* black) showed the lowest concentration, while CH-05 (*P. vulgaris,* black) showed the highest concentration. Further, the oligosaccharides values ranging from 2.70 ± 0.04 to 4.48 ± 0.04 g per 100 g of bean flour were obtained (Table 3). The accessions CH-04 (*P. vulgaris,* red) and CH-13 (*P. vulgaris,* black) showed the lowest and highest contents, respectively.

The lectin activity ranged from 1.50 to 4.46 HAU (hemagglutination unit) per mg protein; CH-16 (*P. coccineus,* purple) showed the lowest lectin activity, and CH-12 (*P. vulgaris,* brown) showed the highest lectin activity. The trypsin inhibition activity ranged from 10.02 ± 0.25 to 16.16 ± 0.46 TIU per g of bean flour; accessions CH-02 (*P. vulgaris,* black) and CH-06 (*P. polyanthus,* yellow) exhibited the lowest and highest trypsin inhibition activity, respectively. 

**Figure 5:**
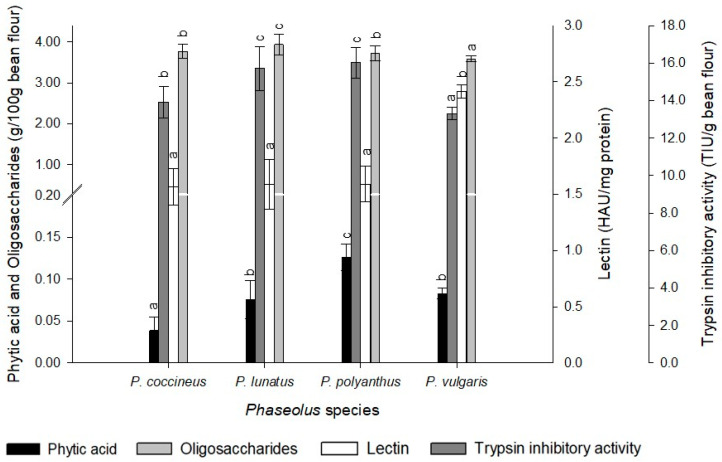
Antinutritional components of bean varieties by *Phaseolus* species. Mean values ± SD with different letters indicate statistically significant differences according to Tukey’ test (*p* < 0.05).

Interspecies analysis indicated that *P. coccineus* had the lowest phytic acid and lectin content, an intermediate value in trypsin inhibitory activity and oligosaccharide. *P. lunatus* showed an intermediate value of phytic acid, together with *P. polyanthus*, the highest trypsin inhibitory activity, and the highest oligosaccharides content of the four species analyzed. *P. polyanthus* was distinguished by the highest phytic acid content. While, *P. vulgaris* exhibited the lowest trypsin inhibitory activity, the highest lectin activity, and the lowest oligosaccharide content than the rest of the *Phaseolus* species (Figure 5).

### 2.6. Discriminant Analysis

**Figure 6:**
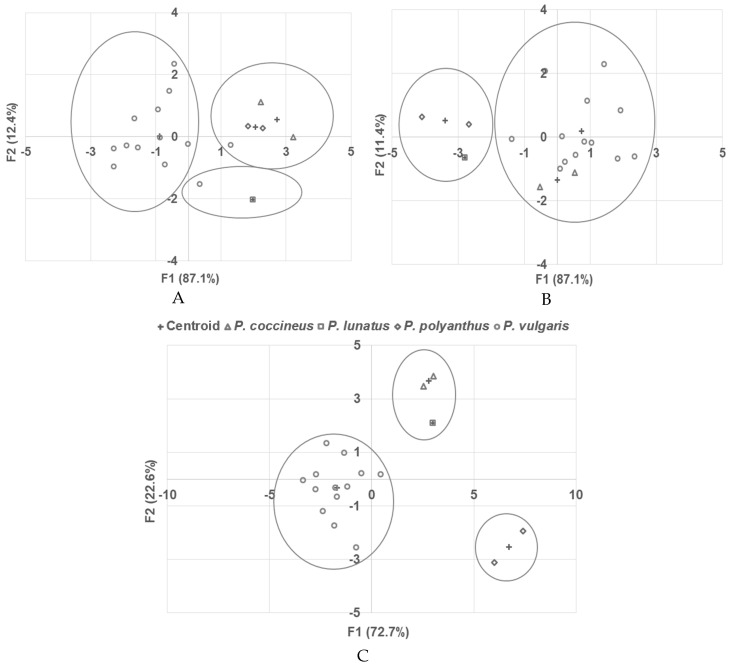
Discriminant analysis of the *Phaseolus* species collected in the Mayan region: (**A**) Phenolic compounds and antioxidant activity, (**B**) antinutritional components, and (**C**) phenolic compounds, antioxidant activity, protein content, and antinutritional components.

Additionally, a discriminant analysis was performed with the phenolic compound concentration and antioxidant activity and showed that the accessions were correctly grouped 83.3% of the time. Only two accessions of *P. vulgaris* exhibited atypical behavior. These samples were CH-12, which was grouped into *P. polyanthus*, and CH-01, which was grouped close to *P. lunatus* (Figure 6a). 

Discriminant analysis from the antinutritional compounds showed that the samples were correctly grouped 88.9% of the time (Figure 6b), and only two atypical samples were observed: CH-01 (*P. vulgaris*, red), which was grouped with *P. lunatus,* and sample CH-14 (*P. vulgaris,* red), which was grouped with the species *P. coccineus*.

Both discriminant analyses between phenolic compounds with antioxidant activity and antinutritional components were performed separately, and atypical behavior was observed in some accessions. However, combining all the variables described above, the accessions were correctly grouped 100% of the time. The closest varieties to each other were *P. coccineus* and *P. lunatus* (Figure 6c). 

## 3. Discussion

In this work, four bean species (*P. vulgaris*, *P. coccineus*, *P. polyanthus*, and *P. lunatus*) were tested. The results are consistent with another research, where it was observed that dark-colored seeds present the lowest luminosity and chromaticity [3]. Therefore, the dimensions collected from the *P. vulgaris* accessions (Table 2) could be considered within the ranges established for the bean races that have been reported to be domesticated in Mesoamerica [12].

However, the *P. lunatus* accession collected is similar in weight to the varieties that belong to the Andean germplasm [13]; the above might be due to the proximity of the Chiapas state with the countries in Central America, where this species is one of the most cultivated. Moreover, for *P. coccineus,* both species collected, according to the morphological analysis, would be classified as medium according to their length [14], although in terms of their weight, they would be classified as large varieties (Table 2).

Along with the purple color of the seed coat, it could be assumed that the accessions collected were more similar to the Andean germplasm, such as *P. lunatus*, which might be since the Mayan region encompasses other Central American countries [12,13]. This indicated that the different species of beans consumed in Chiapas were cultivated and spread throughout the Mayan territory. In the case of *P. polyanthus,* the data coincide with previous research [15], and the accessions collected were within the range of large seeds.

The protein content in legumes ranges from 17 to 40% [16]. In the particular case of common beans (*P. vulgaris*), the protein content depends on the type of bean, and ranges from 14 to 33% are reported. Additionally, the protein is enriched with amino acids such as lysine, phenylalanine, and tyrosine [2]. In the present work, it was found that the CH-09 accession (*P. vulgaris*) showed the highest protein content (30.6%), while the lowest content (19.5%) was found in sample CH-16 (*P. coccineus*). The protein content of CH-09 is higher than that reported in other Mexican varieties of common beans, where the maximum protein content is 26% [2]. Meanwhile, the CH-16 accession has 2.4% less protein than another purple variety (21.9%) of *P. coccineus* [17]. In addition to the variation between species and between varieties of the same species, other factors such as fertilizer use, agricultural practices, and soil-climate characteristics of cultivation sites can influence the protein content of bean seeds [17,18].

Cluster analysis between the species (Figure 2), based on the number of free amino acids, revealed a closeness between *P. vulgaris* and *P. polyanthus*, as well as between *P. coccineus* and *P. lunatus*, with arginine being the predominant amino acid in all species studied. Additionally, arginine was the major amino acid in all the accessions collected. Therefore, the accessions of beans collected could help to prevent the risk of vascular and heart diseases, improve the immune response and renal function, and stimulate the release of pancreatic insulin [19]. Thus, the accessions collected showed potential to produce a nutraceutical agent. 

Phenolic compounds, including simple molecules such as phenolic acids and flavonoids, which are compounds with intermediate molecular weights, and tannins, with high molecular weights, were extracted and quantified for their total phenolic compounds, flavonoids and condensed tannins with different assays. The solvent mix extraction used was adequate since the concentrations reached in each assay were equal to or even higher than those reported in the literature [20]. 

The highest concentration of phenolic compounds and antioxidant activity were found in *P. vulgaris* accessions with red and black seed coats (Figure 3). These results were consistent with another study, who found a positive correlation between total polyphenols and anthocyanins and their antioxidant activity from different varieties of *P. vulgaris* relative to their color [8]. In addition, the accessions collected from *P. coccineus, P. lunatus,* and *P. polyanthus* could reach equal or even higher values of phenolic compounds and antioxidant activity than the accessions from *P. vulgaris* with light colors. Thus, these results showed potential antioxidant activity in all species collected [2].

Furthermore, significant differences were observed between bean species and anthocyanin concentrations. The species that showed significant differences were *P. coccineus* and *P. polyanthus* with *P. lunatus*, and with *P. vulgaris* (Figure 3). Similar results were reported when *P. coccineus* accessions were compared with *P. vulgaris* accessions, where a higher concentration of polyphenols, flavonoids, anthocyanins, and even higher antioxidant activity was found in the accessions exhibiting purple, black, and brown colors [18,21].

In addition, some authors found that the red genotype of different *P. vulgaris* varieties showed significant differences in anthocyanin production among purple- and brown-colored genotypes [22]. Nevertheless, in the present study, the black genotype presented significant differences in the concentration of anthocyanins (*p*-value = 0.002) among the seed coat color of all species. These results were consistent with those reported by Capistrán-Carabarin et al. [21], who found that native beans with black seed coats had the highest content of anthocyanins, even if they were from different species. There is some evidence that has reported the relationship between the anthocyanin and the tannin content and the color of the beans [22]. Thus, this work confirmed the relationship between anthocyanin and tannins with seed color. The black genotype showed a high concentration of both tannins and anthocyanins.

Certain reports have shown that the main flavonoids in some varieties of *P. vulgaris* are quercetin and kaempferol, and both compounds have been shown to decrease the risk of lung cancer and cardiovascular disease [2,3]. In this study, it was observed that kaempferol was only detected in some accessions of *P. vulgaris*, while quercetin was found in almost all the collected accessions, except for the *P. polyanthus* varieties where the presence of this flavonoid was not detected (Figure 4). However, the presence of genistein was observed in both accessions of *P. polyanthus,* at even higher concentrations than those detected in the accessions of *P. coccineus* and *P. vulgaris.* This isoflavonoid is characterized as a phytoestrogen compound that inhibits carcinogenic cells, including breast and prostate cancer, and has until now only been reported in some varieties of *P. vulgaris* during the germination stage, as well as in soybeans [23]. This could suggest that bean accessions collected in the Mayan region of Mexico have high potential to be consumed and improve human health by reducing the risk of some types of cancer.

Regarding ferulic acid content, *P. lunatus* presented an average concentration higher than the accessions of *P. vulgaris* collected (Figure 4). Therefore, this accession may have high antioxidant potential. In the case of anthocyanins, the present work quantified cyanidin 3-glucoside, which was only observed in some varieties of *P. vulgaris* (CH-05, CH-10, and CH-13). This confirms that the composition of anthocyanins in bean seeds can vary, even if the varieties have the same color and that the anthocyanin content depends on both external (climatic and soil conditions) and internal (genetic variation) factors [8,24].

In this sense, some studies have indicated that different species of legumes have a wide variation in their capacity to absorb, translocate, and store flavonoids, polyphenols, and anthocyanins, and together with their variable interaction with cellulose and pectin, this greatly limits the bioavailability of phenolic compounds. These interactions cause great diversity of the compounds present in the bean seeds [21,25]. Finally, it was observed that the phenolic compounds detected have some hydroxyl groups. Hence, it could be assumed that the greater the number of hydroxyl groups, the greater the antioxidant activity [26].

According to correlation analysis (Table 4), the antioxidant activity with the DPPH assay showed a strong positive relationship with flavonoids (r = 0.95) and anthocyanins (r = 0.96). These correlations could be related to the extraction system used, since there is some evidence that acetone solvent extraction exhibits a higher yield of flavonoids and ethanol solvent extraction exhibits a higher yield of anthocyanins [20,27]. Furthermore, some reports point out that the interaction between the compounds with antioxidant potential and the DPPH* radical is affected by the medium where these compounds were extracted [20]. Thus, the antioxidant activity depends not only on the concentration of phenolic compounds but also on the polarity and chemical structure of each phenolic compound [2,28]. Therefore, it could be assumed that the mechanism of interaction between the antioxidant potential of collected beans and the DPPH* radical is more structurally related and responds faster, reducing the number of DPPH* molecules in the medium [29].

Among the antinutritional compounds, reported in beans, are phytic acid, trypsin inhibitory activity, lectins and oligosaccharides, where significant differences were found among the four species evaluated. However, recent studies have reported that trypsin inhibitors and lectins could confer some health benefits. For example, trypsin inhibitors could confer protection against rotavirus, inhibit some types of carcinogenesis, and could be used as chemopreventive agents [10], and lectins may decrease lymphoma growth and could be used as diagnostic markers for tumors, as well as help in the prevention of obesity [2].

The antinutritional compounds were determined in raw bean seeds, therefore it is important to mention that there are several methods and processing techniques to reduce the levels of these antinutritional factors. Treatments such as soaking, milling, boiling, microwave cooking, autoclaving, hydrothermal processing, and germination, either individually or in combination, can inhibit or reduce the tannin content, trypsin inhibitor activity, phytic acids and hemagglutinins [30]. 

In this work, the results showed a positive correlation (r = 0.97) between lectins and antioxidant activity (DPPH assay), where accessions CH-16 (*P. coccineus*) and CH-12 (*P. vulgaris*) presented the lowest and highest contents, respectively. Therefore, it could be assumed that there is a relationship between lectins and antioxidant activity that could help prevent some types of diseases related to inflammation [31]. Thus, it could be confirmed that the accessions collected have a high potential to be used as precursors for nutraceutical products due to their chemical composition and high nutritional content.

Taking into account all the results, the bean species collected in the Mayan region of Mexico exhibit high potential for biological activity that could be used to develop products due their nutritional, phenolic content, and their antioxidant effect could prevent some chronic diseases related to the oxidative stress [32].

## 4. Materials and Methods 

### 4.1. Plant Material

Different bean accessions were collected in various rural communities in the Mayan region of Chiapas State, Mexico, in March 2019 (Table 5). The accessions were classified according to their physical and morphological characteristics within the bean species *P. vulgaris*, *P. coccineus*, *P. lunatus*, or *P. polyanthus*. These species are located in the Mayan region, particularly in the State of Chiapas, which is characterized by great agrodiversity. In this region, rural areas with low economic resources, a lack of social assistance and information on health issues, low academic preparation, and the deep-rooted predominance of their culture, among other factors, influence the nutritional status of these communities [33].

### 4.2. Physicochemical Analysis

Once the plant material was collected, the bean seeds were washed to remove any foreign material and dried in a convection oven for 4 h at 40 °C (Convection oven plus HCX II, SAN-SON, Mexico). Afterwards, the seeds were ground to fine powder and kept in hermetically sealed containers for further determination of phenolic compounds, antioxidant activity, and content of antinutritional factors.

#### 4.2.1. Bean Size Determination

One hundred bean seeds of each accession were randomly measured (length, with, and thickness) and weighed to determine if the seeds were small (<25 g), medium (25–40 g) or large (>40 g) [13].

#### 4.2.2. Bean Color Evaluation

These parameters were determined according to the CIE L*A*B* scale using a spectrophotometer (CM-5, Konica Minolta Sensing Americas, Ramsey, NJ, USA) and were calculated according to the following equations: Brightness (L) with a range of 0 (black) to 100 (white), chromaticity (c) and hue (h); these last two parameters were calculated according to the following equations:

c = (a^*2^ + b^*2^)^1/2^ (1)

h = tan^−1^(b*/a*) (2)

where a* = this variable being positive represents red tones and this value being negative represents green tones; b* = this variable being positive represents yellow tones and this variable being negative represents blue tones [11].

#### 4.2.3. Proximate Composition Analysis of Bean Seeds

This analysis was performed according to the AOAC international official methods of analysis: protein (960.52), moisture (945.38), ash (930.03), lipids (920.30), raw fiber (985.28), and total carbohydrates by difference from the proximate composition [34].

#### 4.2.4. Total Amino Acid Analysis

The following amino acids were quantified based on the official method 982.30 (a) of AOAC: Histidine (His), arginine (Arg), threonine (Thr), lysine (Lys), valine (Val), isoleucine (Ile), leucine (Leu), phenylalanine (Phe), methionine (Met), and tryptophan (Trp) [35].

### 4.3. Phenolic Compounds

#### 4.3.1. Extraction of Phenolic Compounds

Bean flour of the 18 accessions was obtained by milling (A11 mill, IKA, Staufen, Germany) and passing through MESH 60. Later, maceration was carried out at room temperature from 16 h by weighing 15 g of bean flour (dry weight, dw) and 150 mL of acetone solvent mixture (acetone, water, and acetic acid, 70:29.5:05 *v*/*v*/*v*). Then, the extracts were centrifuged at 3488× *g* for 15 min (SL 40R, Thermo Scientific, Waltham, MA, USA), washed with the solvent mixture once, and the supernatant was retained. Finally, the extract was concentrated using a rotary evaporator at 45 °C and 450 mbar (R-210, BUCHI, Flawil, Switzerland). The extracts were frozen at −20 °C until analysis [13,20].

#### 4.3.2. Determination of Total Phenols

Folin–Ciocalteu (F–C) spectrophotometric method was performed [16]. Quantification of the phenolic compounds was performed with gallic acid (mg GAE/g dw) as follows: 50 µL of each sample was mixed in 3 mL of distilled water, and then 250 µL of F–C reagent (2N) and 750 µL of 7% (*w*/*v*) sodium carbonate were added. Later, the mixture was allowed to incubate for 8 min at room temperature (RT). Finally, 950 µL of distilled water was added, followed by homogenization and incubation in the dark for 2 h at RT, and the absorbance was measured at 765 nm (Infinite M200 Pro, TECAN, Männedorf, Switzerland).

#### 4.3.3. Determination of Flavonoids

Aluminum chloride (AlCl_3_) test was carried out [16,18]. Quantification of the flavonoid content was expressed as quercetin equivalents per gram of bean flour (mg QE/g dw) by mixing 50 µL of each sample, 700 µL of deionized water, and 250 µL of AlCl_3_ [133 mg of AlCl_3_ and 400 mg of sodium acetate in 100 mL of methanol-water-acetic acid (140:50:10 *v*/*v*/*v*)]. Then, the solution was mixed and kept at RT for 30 min for completion of the reaction. Finally, the absorbance was measured at 410 nm.

#### 4.3.4. Determination of Condensed Tannins

Vanillin test was used to quantify the condensed tannins based on the catechin equivalents per gram of bean flour (mg CAE/g dw). Briefly, 500 µL of each sample, 3 mL of 4% (*w*/*v*) vanillin methanol solution, and 1.5 mL of 50% (*v*/*v*) sulfuric acid solution were mixed, gently stirred and incubated at RT for 15 min in the dark [36,37]. Later, the absorbance was measured at 550 nm.

#### 4.3.5. Determination of Anthocyanins

This analysis was carried out from the bean seed coat using AOAC method 2005.02 [6]. One hundred milligrams of bean seed coat was weighed and extracted with 2.4 mL of acidified ethanol (1N HCl, 85:15 *v*/*v*) for 15 min. Then, the volume of the samples was adjusted to 5 mL with acidified ethanol and centrifuged at 3488× *g* for 15 min, and the supernatant was recovered to carry out the corresponding analyses. The absorbance at 520 and 700 nm was measured with a spectrometer (Infinite M200 Pro, TECAN, Männedorf, Switzerland). Finally, the following equation was used to calculate the concentration of anthocyanins as mg of cyanidin 3-glucoside per liter.

(3) $$\frac{\mathrm{EC}3G}{L}=\frac{\left[{\mathrm{pH}}_{1}\left(A_{520\;\mathrm{nm}}-A_{700\;\mathrm{nm}}\right)-{\;\mathrm{pH}}_{4.5}\left(A_{520\;\mathrm{nm}}-A_{700\;\mathrm{nm}}\right)\times 449.2\times \mathrm{DF}\;\times 1000\right]}{\left(26900\times D\right)}$$

where DF = Dilution factor, D = 1, 449.2 = molecular weight of cyanidin 3-glucoside, and 26,900 = molar extinction coefficient.

#### 4.3.6. Quantification of Phenolic Compounds by UHPLC

Bean flour was weighed at 0.5 g and extracted into 10 mL of ethanol with 1% trifluoroacetic acid (TFA) and refrigerated at 4 °C for 24 h. Then, the samples were centrifuged (3,000 rpm) for 5 min, and the supernatant was collected. Finally, the residue was washed once and centrifuged under the same conditions; both supernatants were mixed for analysis by liquid chromatography (UHPLC).

UHPLC analysis was performed with a PDA Detector (Acquity Arc, Waters, Milford, MA, USA), and the gradient conditions started with 90% solvent A, followed by 82% A at 10 min, 72% A at 18 min, 60% A at 19 min, and 90% A at 23 min. Solvent A was water with 0.1% TFA, and solvent B was acetonitrile with 0.1% TFA, with a flow rate of 0.7 mL/min. The column used was a C18, 2.7 μm, 4.6 × 150 mm (Cortects, Waters, Milford, MA, USA), the oven was set at 30 °C, and the volume injected was 20 μL. The compounds quantified in this study were detected at different wavelengths; ferulic acid was analyzed at 250 nm, kaempferol 3-glucoside, quercetin 3-glucoside, and genistein were analyzed at 300 nm, and cyanidin 3-glucoside was analyzed at 550 nm. All samples were injected in duplicate [24].

### 4.4. Determination of Antioxidant Activity

#### 4.4.1. ABTS Assay (2,2′-Azino-bis (3-ethylbenzothiazoline-6-sulfonic acid) Diammonium Salt)

This analysis was performed by mixing 20 µL of each sample and 270 µL of the ABTS^+^ radical. A solution of 7 mM ABTS and 2.5 mM potassium persulfate was used, and the reaction was allowed to stabilize for 12 to 16 h in the dark at RT. The ABTS^+^ solution was diluted in ethanol until an absorbance of 0.70 ± 0.02 was obtained at 734 nm (Infinite M200 Pro, TECAN, Männedorf, Switzerland). Reaction blanks were prepared with an ethanol solution and the control sample was deionized water (20 µL) and the ABTS^+^ radical solution (270 µL). The absorbance was measured at 734 nm, 6 min after the initial mixing until the reaction was complete, which was 24 min after starting the reaction [28].

#### 4.4.2. DPPH Assay (2, 2 Diphenyl-1-picrylhydrazyl)

This test was carried out by mixing 100 µL of each sample and 100 µL of the DPPH* radical. The absorbance was measured at 515 nm every 5 min after the beginning of the reaction until 30 min had elapsed [28].

Both ABTS^+^ (ARSA %) and DPPH* (DRSA %) radical scavenging activity was measured following this equation:

(4) $$\mathrm{ARSA}\;\left(\%\right)\;\mathrm{or}\;\mathrm{DRSA}\;\left(\%\right)\;\mathrm{activity}=\left[1-\left(\frac{A_{m-}A_{b}}{A_{c}}\right)\right]\times 100$$

where Am = sample absorbance (sample and ABTS^+^ radical solution or sample and DPPH* radical solution), Ab = blank absorbance (sample and ethanol or sample and methanol), Ac = control absorbance (deionized water and ABTS+ radical solution or deionized water and DPPH* radical solution).

### 4.5. Antinutritional Compounds

#### 4.5.1. Phytic Acid Assay

This assay was performed enzymatically from bean flour, using the commercial phytic acid (phytate)/total phosphorus Megazyme kit (K-PHYT, Megazyme, Wicklow, Ireland), according to the instructions provided by the supplier.

#### 4.5.2. Oligosaccharide Assay

This assay was carried out enzymatically from bean flour, using the raffinose/sucrose/glucose Megazyme kit (K-RAFGL, Megazyme, Wicklow, Ireland), following the instructions provided by the supplier.

#### 4.5.3. Determination of Lectin Activity

The bean flour extract used to determine lectin activity (LA) was prepared following the methodology proposed by previous authors [38], with some modifications. Phosphate buffer (PBS, pH 7.4) was used as a buffer solution with the flour in a ratio of 1:13 (*w*/*v*). The solution was left under continuous stirring for 18 h. The mixture was centrifuged at 12,000× *g* for 20 min, obtaining the supernatant for the agglutination test.

The erythrocyte preparation was carried out with human blood type A^+^ in a heparin tube, taking 1 mL of blood and adding 1 mL of PBS. The erythrocytes were washed twice with the same solution and centrifuged (847× *g* for 8 min). Once the erythrocytes were obtained, a 4% (*v*/*v*) solution was prepared.

The agglutination test was carried out in 96-well ELISA plates, in which 50 µL of PBS and 50 µL of the bean flour extract sample were placed, and two-fold dilutions were made. Afterward, 50 µL of erythrocyte solution was added to each well and the mixture was allowed to stand at controlled RT for 1 h.

Controls were a 0.2% *Phaseolus vulgaris* L. lectin solution, used as a positive control, and a PBS solution, used as a negative control. The lectin activity was expressed as the agglutination activity, which was the inverse of the maximum dilution at which agglutination was observed in mg of protein (HAU/mg protein).

#### 4.5.4. Determination of Trypsin Inhibitory Activity

Preparation of the bean sample was performed according to Kakade et al. [39]. One gram of raw bean flour was weighed, and 50 mL of sodium hydroxide (NaOH) was added followed by stirring for 3 h to ensure maximum extraction. Then, the solution was adjusted to pH 8.2 and centrifuged at 3,000× *g* for 10 min, and the precipitate was discarded.

The trypsin activity methodology was performed using the commercial trypsin activity colorimetric kit (MAK290-1KT, Sigma-Aldrich, St. Louis, MO, USA), with some modifications. The reagents were prepared according to the instructions of the kit, where the standard curve of p-nitroaniline (p-Na) was used at five concentrations (2, 4, 6, 8, and 10 nM). The positive control with trypsin binds to the substrate to generate p-Na, and the color intensity is proportional to the content of p-Na, allowing for measurement of the trypsin activity. Bean samples containing trypsin inhibitors may inhibit the formation of p-Na. One microliter of trypsin solution was added to the samples. The control was the sample without the addition of any inhibitor (100% trypsin formation).

Trypsin activity was calculated, and trypsin activity formation was compared with the control to determine the TIU, which is the amount of inhibitor that is required to inhibit one microgram of pure trypsin. The results are reported as TIU per g of sample.

### 4.6. Statisdical Analysis

Statistics performed include the average, standard deviation, and analysis of variance (ANOVA) with a confidence level of α = 0.05. In addition, correlation studies were performed with Pearson’s correlation coefficients and discriminant and cluster analyses to reveal the relationship and differences between the species of beans collected. To assess the difference between mean values, Tukey’s test was performed with a *p* < 0.05 significance level. The statistical software Statgraphics Centurion XVI (Version 16.1.03) (Statgraphics Technologies, The Plains, VA, USA) was used.

## 5. Conclusions

In all species collected, a strong correlation was obtained between the antioxidant activity (DPPH) and the content of flavonoids, anthocyanins, and lectin. Similarly, quercetin 3-glucoside and genistein were the main phenolic compounds found in almost all the accessions analyzed.

Furthermore, *P. vulgaris* accessions showed the highest concentration of phenolic compounds and antioxidant activity. Two accessions of *P. vulgaris* (CH-01 and CH-12) showed a similar composition of phenolic compound content and antioxidant activity as the accessions of *P. lunatus* and *P. polyanthus*. Moreover, two accessions of *P. vulgaris* (CH-01 and CH-14) presented similar compositions of antinutritional content and could be grouped with the accessions of *P. lunatus* and *P. coccineus*. However, it was observed that there is a clear division between species (100%) based on their phenolic compounds, protein content, antioxidant activity, and antinutritional components. Therefore, it can be assumed that accessions of different bean species have a high biological potential for the prevention of diseases such as cancer, heart disease, the immune system, and obesity, among others. In addition, this study aimed to encourage and expand human consumption of bean species that are currently underutilized, as well as to promote their production and conservation.

## Figures and Tables

**Table 1:** Luminosity (*L), chromaticity (c), hue (h) of the grain beans and physical characteristics of the different species of *Phaseolus*.

Sample	*L	c	h	Length (mm)	Width (mm)	Thickness (mm)	Weigh (g)	Seed Coat Color
CH-01 *P*.*v*.	27.97 ± 3.32 ^b–d^	20.89 ± 0.12 ^e,f^	0.50 ± 0.02 ^b,c^	14.4 ± 1.2 ^e–g^	5.9 ± 0.5 ^a–c^	4.3 ± 0.5 ^a–d^	43.4 ± 0.4 ^c–e^	Red
CH-02 *P*.*v*.	15.01 ± 0.34 ^a^	1.92 ± 0.05 ^b^	1.22 ± 0.05 ^c,d^	9.2 ± 1.1 ^a,b^	4.9 ± 0.6 ^a^	3.2 ± 0.5 ^a^	18.6 ± 0.6 ^a^	Black (violet)
CH-03 *P*.*v*.	17.50 ± 0.44 ^a^	1.21 ± 0.66 ^a,b^	−1.48 ± 0.08 ^a^	8.4 ± 1.1 ^a^	5.0 ± 0.6 ^a^	3.3 ± 0.4 ^a,b^	20.2 ± 0.2 ^a^	Black (blue)
CH-04 *P*.*v*.	27.37 ± 0.19 ^bc^	20.02 ± 0.20 ^e^	0.55 ± 0.01 ^b,c^	9.4 ± 0.9 ^a,b^	5.7 ± 0.5 ^a,b^	4.5 ± 0.6 ^a–e^	31.0 ± 0.5 ^a–c^	Red
CH-05 *P*.*v*.	17.73 ± 0.26 ^a^	0.11 ± 0.06 ^a^	0.58 ± 0.78 ^b,c^	11.5 ± 1.2 ^b–e^	6.1 ± 0.6 ^a–d^	3.7 ± 0.6 ^a–c^	29.3 ± 0.9 ^a,b^	Black (violet)
CH-06 *P*.*p*.	23.75 ± 0.71 ^b^	28.47 ± 0.77 ^h^	0.73 ± 0.01 ^b–d^	12.9 ± 1.1 ^c–f^	10.1 ± 0.9 ^g^	6.5 ± 0.7 ^g–i^	81.9 ± 0.4 ^f,g^	Yellow
CH-07 *P*.*c*.	26.51 ± 0.45 ^b,c^	11.92 ± 0.16 ^c^	−0.06 ± 0.06 ^b^	16.3 ± 1.4 ^g^	10.5 ± 1.0 ^g^	5.8 ± 0.8 ^e–h^	88.5 ± 1.3 ^g^	Purple
CH-08 *P*.*v*.	69.43 ± 0.81 ^g^	14.71 ± 0.12 ^d^	1.48 ± 0.01 ^d^	10.3 ± 1.2 ^a–c^	6.4 ± 0.6 ^a–e^	4.7 ± 0.7 ^b–f^	37.2 ± 0.7 ^b–d^	White
CH-09 *P*.*v*.	24.39 ± 0.16 ^b^	22.13 ± 0.62 ^f,g^	0.48 ± 0.01 ^b,c^	8.6 ± 0.7 ^a^	4.7 ± 0.6 ^a^	3.5 ± 0.4 ^a,b^	20.4 ± 0.2 ^a^	Red
CH-10 *P*.*v*.	16.12 ± 0.32 ^a^	0.91 ± 0.10 ^a,b^	−1.47 ± 0.05 ^a^	12.6 ± 1.2 ^c–e^	7.9 ± 0.7 ^d,e^	4.6 ± 0.5 ^a–f^	40.9 ± 3.3 ^b–e^	Black (blue)
CH-11 *P*.*p*.	18.89 ± 0.12 ^a^	23.13 ± 0.41 ^g^	0.59 ± 0.0 1 ^b,c^	13.4 ± 1.1 ^d–f^	10.5 ± 0.7 ^g^	7.4 ± 0.6 ^i^	73.0 ± 2.0 ^f^	Red
CH-12 *P*.*v*.	39.06 ± 0.36 ^e^	21.89 ± 0.07 ^f,g^	1.09 ± 0.01 ^c,d^	10.3 ± 0.8 ^a–c^	8.0 ± 0.7 ^e,f^	6.1 ± 0.9 ^f–i^	43.3ׅ ± 3.1 ^c–e^	Brown
CH-13 *P*.*v*.	15.30 ± 0.38 ^a^	0.88 ± 0.13 ^a,b^	−1.43 ± 0.03 ^a^	10.7 ± 0.8 ^a–d^	6.1 ± 0.5 ^a–d^	4.5 ± 0.4 ^a–e^	21.1 ± 0.1 ^a^	Black (blue)
CH-14 *P*.*v*.	25.53 ± 0.16 ^b^	20.06 ± 0.35 ^e^	0.37 ± 0.01 ^b,c^	10.1 ± 0.8 ^a–c^	7.4 ± 0.6 ^b–e^	5.8 ± 0.5 ^d–h^	30.5 ± 0.2 ^a,b^	Red
CH-15 *P*.*v*.	15.79 ± 0.44 ^a^	0.76 ± 0.12 ^a,b^	−1.44 ± 0.02 ^a^	10.7 ± 0.9 ^a-d^	7.2 ± 0.5 ^b–e^	5.1 ± 0.4 ^c–g^	28.3 ± 03 ^a,b^	Black (blue)
CH-16 *P*.*c*.	30.62 ± 0.08 ^c,d^	13.80 ± 0.06 ^d^	0.04 ± 0.01 ^b^	15.6 ± 1.3 ^f,g^	11.0 ± 1.0 ^g^	6.9 ± 0.7 ^h,i^	75.7 ± 1.9 ^f^	Purple
CH-17 *P*.*l*.	31.92 ± 0.32 ^d^	28.33 ± 0.22 ^h^	0.55 ± 0.01 ^b,c^	13.7 ± 1.1 ^e–g^	9.8 ± 0.6 ^f,g^	4.5 ± 0.3 ^a–e^	44.9 ± 0.2 ^d,e^	Pink
CH-18 *P*.*v*.	49.23 ± 1.26 ^f^	21.63 ± 0.42 ^e–g^	1.00 ± 0.02 ^c,d^	16.4 ± 1.0 ^g^	7.6 ± 0.4 ^c–e^	5.9 ± 0.4 ^e–i^	50.8 ± 0.5 ^e^	Brown

Mean values ± SD with different lowercase letters within the same column denote significant differences based on Tukey’s test (*p* < 0.05). Abbreviations: *P*.*v*. = *Phaseolus vulgaris*, *P*.*p*. = *P*. *polyanthus*, *P*.*c*. = *P*. *coccineus*, *P*.*l*. = *P*. *lunatus*.

**Table 2:** Free amino acid content from *Phaseolus* species grown in the Mayan Region.

Amino Acid (mg/100 g Dry Beans)	*Phaseolus* Species			
	*P. coccineus*	*P. lunatus*	*P. polyanthus*	*P. vulgaris*
Histidine	10.66 ± 0.57 ^a^	14.02 ± 0.00 ^a^	24.08 ± 0.32 ^b^	40.08 ± 0.39 ^c^
Arginine	157.71 ± 0.38 ^a^	127.88 ± 0.00 ^a^	151.84 ± 0.27 ^a^	380.98 ± 0.39 ^b^
Threonine	15.32 ± 0.62 ^a^	15.36 ± 0.0 ^a,b^	28.22 ± 0.51 ^c^	19.08 ± 0.42 ^b^
Lysine	14.33 ± 0.63 ^a,b^	12.80 ± 0.00 ^a^	14.84 ± 0.49 ^a,b^	16.70 ± 0.37 ^b^
Valine	15.18 ± 0.62 ^a,b^	14.84 ± 0.00 ^a,b^	27.20 ± 0.52 ^c^	19.07 ± 0.42 ^b^
Isoleucine	4.51 ± 0.59 ^a^	5.27 ± 0.00 ^a^	8.70 ± 0.67 ^c^	7.24 ± 0.46 ^b^
Leucine	12.72 ± 0.14 ^a^	19.09 ± 0.00 ^b^	20.01 ± 0.09 ^b^	19.18 ± 0.18 ^b^
Phenylalanine	9.14 ± 0.62 ^a,b^	8.68 ± 0.00 ^a,b^	12.08 ± 0.68 ^b^	10.51 ± 0.48 ^a,b^
Methionine	1.75 ± 0.76 ^a^	2.35 ± 0.00 ^a,b^	2.56 ± 0.91 ^a^	3.34 ± 0.57 ^b^
Tryptophan	14.13 ± 0.38 ^a^	12.77 ± 0.00 ^a^	15.14 ± 0.30 ^a^	48.19 ± 0.53 ^b^

Mean values ± SD with different lowercase letters within the same row denote significant differences based on Tukey’s test (*p* < 0.05).

**Table 3:** Total phenolic (TPH), flavonoid (FLV), anthocyanin (ANT), tannin (TAN), phytic acid (PHY), oligosaccharide (OSD), lectin (LET), trypsin inhibitory activity (TRY) content, and radical scavenging activity (%ARSA, %DRSA) from *Phaseolus* accessions grown in the Mayan Region.

Sample	TPH	FLV	ANT	TAN	PHY	OSD	LET	TRY	%ARSA	%DRSA
CH-01	1043.3 ± 13.2 ^d,e^	5.7 ± 0.2 ^a^	0.69 ± 0.06 ^a^	1.42 ± 0.08 ^a,b^	96.1 ± 0.5 ^a–e^	3.21 ± 0.17 ^a–c^	1.74	14.7 ± 0.5 ^d–g^	90.9 ± 0.0 ^a,b^	92.0 ± 0.0 ^c–f^
CH-02	661.2 ± 27.8 ^b–d^	6.4 ± 0.3 ^a,b^	3.60 ± 0.16 ^b^	1.62 ± 0.11 ^a,b^	76.1 ± 0.6 ^a–e^	2.96 ± 0.07 ^a,b^	2.05	10.02 ± 0.25 ^a^	61.9 ± 0.3 ^a,b^	91.8 ± 0.0 ^c–f^
CH-03	550.1 ± 0.5 ^a–c^	5.9 ± 0.2 ^a^	2.82 ± 0.06 ^b^	3.83 ± 0.04 ^d,e^	49.1 ± 0.5 ^a–d^	3.00 ± 0.04 ^a,b^	1.62	15.6 ± 2.3 ^e–g^	80.5 ± 0.2 ^a,b^	94.0 ± 0.1 ^e,f^
CH-04	861.1 ± 0.8 ^c,d^	11.7 ± 0.1 ^a–c^	0.50 ± 0.05 ^a^	3.60 ± 0.04 ^d^	144.7 ± 0.3 ^d,e^	2.70 ± 0.04 ^a^	2.00	13.0 ± 0.7 ^b–e^	56.7 ± 0.1 ^a^	92.5 ± 0.0 ^d–f^
CH-05	607.6 ± 0.9 ^b–d^	7.6 ± 0.2 ^a–c^	6.22 ± 0.05 ^c^	5.00 ± 0.03 ^e,f^	158.2 ± 0.5 ^e^	3.47 ± 0.05 ^b–e^	1.88	14.3 ± 0.9 ^c–g^	96.3 ± 01 ^b^	95.0 ± 0.0 ^f^
CH-06	660.5 ± 21.9 ^b–d^	1.7 ± 0.2 ^a^	0.19 ± 0.05 ^a^	0.87 ± 0.02 ^a^	130.2 ± 0.2 ^c–f^	4.44 ± 0.03 ^g,h^	1.59	16.2 ± 0.5 ^g^	63.6 ± 0.1 ^a,b^	84.2 ± 0.0 a^,b^
CH-07	627.4 ± 3.5 ^b–d^	1.8 ± 0.1 ^a^	0.26 ± 0.23 ^a^	1.34 ± 0.02 ^a,b^	29.8 ± 0.6 ^a,b^	3.86 ± 0.08 ^d–h^	1.63	14.3 ± 0.1 ^c–g^	57.4 ± 0.2 ^a^	85.3 ± 0.0 ^a–d^
CH-08	114.9 ± 0.7 ^a^	1.1 ± 0.3 ^a^	ND	0.53 ± 0.06 ^a^	114.2 ± 0.9 ^a–e^	3.83 ± 0.13 ^d–g^	2.12	11.5 ± 0.1 ^a,b^	89.5 ± 0.1 ^a,b^	85.6 ± 0.0 ^a–d^
CH-09	1364.5 ± 0.5 ^e^	19.9 ± 0.5 ^c^	0.59 ± 0.04 ^a^	3.40 ± 0.02 ^c,d^	34.9 ± 0.8 ^a–c^	4.37 ± 0.07 ^g,h^	2.36	14.2 ± 2.2 ^c–g^	88.9 ± 0.3 ^a,b^	94.6 ± 0.02 ^f^
CH-10	787.5 ± 17.0 ^b–d^	18.6 ± 0.2 ^b,c^	9.42 ± 0.03 ^d^	5.57 ± 0.02 ^f^	24.1 ± 0.4 ^a^	3.74 ± 0.04 ^c–f^	2.12	14.3 ± 0.5 ^c–f^	80.6 ± 0.1 ^a,b^	87.0 ± 0.1 ^b–e^
CH-11	595.8 ± 24.0 ^b–d^	1.6 ± 0.2 ^a^	0.10 ± 0.09 ^a^	0.85 ± 0.01 ^a^	121.8 ± 0.1 ^b–e^	3.01 ± 0.09 ^a,b^	1.59	15.9 ± 0.1 ^f,g^	65.5 ± 0.3 ^a,b^	85.0 ± 0.0 ^a–c^
CH-12	573.8 ± 12.5 ^a–d^	4.2 ± 0.3 ^a^	0.22 ± 0.03 ^a^	0.82 ± 0.06 ^a^	33.2 ± 0.4 ^a,b^	3.40 ± 0.07 ^b–d^	2.10	15.5 ± 1.3 ^e–g^	70.0 ± 0.2 ^a,b^	82.9 ± 0.0 ^a,b^
CH-13	846.5 ± 16.8 ^c,d^	8.7 ± 0.4 ^a–c^	5.70 ± 0.09 ^c^	4.92 ± 0.02 ^e,f^	96.8 ± 0.9 ^a–e^	4.48 ± 0.04 ^h^	1.96	15.5 ± 1.4 ^e–g^	70.0 ± 0.2 ^a,b^	82.9 ± 0.0 ^a,b^
CH-14	343.8 ± 0.9 ^a,b^	5.1 ± 0.2 ^a^	0.43 ± 0.07 ^a^	0.59 ± 0.01 ^a^	64.9 ± 0.4 ^a–e^	4.07 ± 0.05 ^e–h^	1.78	11.9 ± 1.1 ^a–c^	76.7 ± 0.1 ^a,b^	86.5 ± 0.1 ^b–d^
CH-15	567.1 ± 1.9 ^a–d^	5.6 ± 0.1 ^a^	5.30 ± 0.09 ^c^	2.18 ± 0.50 ^b,c^	73.1 ± 0.2 ^a–e^	4.24 ± 0.^05 f–h^	1.68	13.4 ± 0.3 ^b–f^	79.0 ± 0.1 ^a,b^	94.8 ± 0.1 ^f^
CH-16	506.0 ± 18.3 ^a–c^	1.4 ± 0.1 ^a^	0.27 ± 0.07 ^a^	1.70 ± 0.03 ^a,b^	47.2 ± 0.6 ^a–c^	3.69 ± 0.16 ^c–f^	1.50	13.5 ± 1.3 ^b–g^	63.9 ± 0.2 ^a,b^	78.9 ± 0.6 ^a^
CH-17	662.0 ± 20.1 ^b–d^	2.2 ± 0.1 ^a^	0.050 ± 0.14 ^a^	0.90 ± 0.02 ^a,b^	75.3 ± 0.3 ^a–e^	3.94 ± 0.06 ^d–h^	1.59	15.7 ± 0.8 ^f,g^	87.5 ± 0.0 ^a,b^	82.1 ± 0.1 ^a,b^
CH-18	451.9 ± 28.2 ^a–c^	1.2 ± 0.1 ^a^	0.10 ± 0.11 ^a^	0.80 ± 0.04 ^a^	111.4 ± 0.5 ^a–e^	3.18 ± 0.04 ^a–c^	1.54	12.2 ± 1.5 ^a–d^	96.9 ± 0.0 ^b^	98.2 ± 0.4 ^f^

**TPH** = mg GAE/g dw; **FLV** = mg QE/g dw; **ANT** = mg cyaniding 3-glucoside/g seed coat; **TAN** = mg CAE/g dw; **PHY** = mg/100g bean flour; **OSD** = mg/100g bean flour; **LET** = HAU/mg protein; **TRY** = TIU/g bean flour. ND = undetected. Mean values ± SD with different lowercase letters within the same column denote significant differences based on Tukey’s test (*p* < 0.05).

**Table 4:** Pearson’s correlation coefficients of the bioactive compounds of the different *Phaseolus* species.

Correlation	r	*p*-Value
Flavonoids and Anthocyanins	0.9891	0.0109
Flavonoids and %DRSA	0.9525	0.0475
Flavonoids and Lectin	0.9975	0.0025
Tannins and Anthocyanins	0.9511	0.0489
Anthocyanins and %DRSA	0.9609	0.0391
Anthocyanins and Lectin	0.9959	0.0041
%DRSA and Lectin	0.9669	0.0331

DRSA = DPPH radical scavenging activity.

**Table 5:** *Phaseolus* species of beans collected in the Mayan region (Mexico).

Sample	*Phaseolus* Species	Common Name	Origin	Picture
CH-01	*P. vulgaris*	Pinto Colorado (*Tzirin chenek*)	Tenejapa	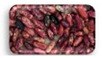
CH-02	*P. vulgaris*	Negro Cubano	Villaflores	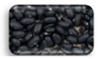
CH-03	*P. vulgaris*	Negro graceño	Villaflores	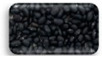
CH-04	*P. vulgaris*	Pie de Paloma	Teopisca	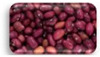
CH-05	*P. vulgaris*	Negro de vaina blanca	Teopisca	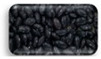
CH-06	*P. polyanthus*	Ibes	Tenejapa	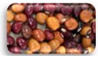
CH-07	*P. coccineus*	Botil	Tenejapa	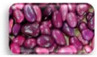
CH-08	*P. vulgaris*	Blanco	Teopisca	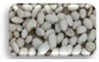
CH-09	*P. vulgaris*	Rojo	Chenalho	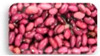
CH-10	*P. vulgaris*	Negro de vaina morada	Teopisca	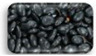
CH-11	*P. polyanthus*	Ibes rojo	Chenalho	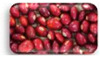
CH-12	*P. vulgaris*	Bayo	Teopisca	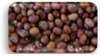
CH-13	*P. vulgaris*	Negro	Chenalho	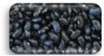
CH-14	*P. vulgaris*	Regadillo	Teopisca	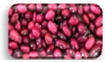
CH-15	*P. vulgaris*	Negro	Pantelho	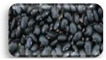
CH-16	*P. coccineus*	Botil	Chamula	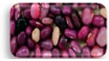
CH-17	*P. lunatus*	Patachete	Teopisca	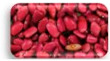
CH-18	*P. vulgaris*	Barreton	Teopisca	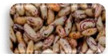

## References

[1] Romero-Arenas O., Damián-Huato M., Rivera-Tapia J.A., Báez-Simón A., Huerta-Lara M., Cabrera-Huerta E. (2013). The nutritional value of Beans (*Phaseolus vulgaris* L.) and its importance for feeding of rural communities in Puebla-Mexico. Int. Res. J. Biological Sci..

[2] Chávez-Mendoza C., Sánchez E. (2017). Bioactive compounds from Mexican varieties of the common bean (*Phaseolus vulgaris*): Implications for health. Molecules.

[3] Espinosa-Alonso L.G., Lygin A., Widholm J.M., Valverde M.E., Paredes-Lopez O. (2006). Polyphenols in wild and weedy Mexican common beans (*Phaseolus vulgaris* L.). J. Agric. Food Chem..

[4] Juárez-López B.A., Aparicio-Fernández X., Nevárez-Moorllón G.V., Ortega-Rivas E. (2012). Polyphenolics concentration and antiradical capacity of common bean varieties (*Phaseolus vulgaris* L.) after thermal treatment. Food Science and Food Biotechnology Essentials: A Contemporary Perspective.

[5] Luthria D.L., Pastor-Corrales M.A. (2006). Phenolic acid content of fifteen dry edible beans (*Phaseolus vulgaris* L.) varieties. J. Food Compos. Anal..

[6] Mojica L., Meyer A., Berhow M.A., González de Mejia E. (2015). Bean cultivars (*Phaseolus vulgaris* L.) have similar high antioxidant capacity, *in vitro* inhibition of α-amylase and α-glucosidase while diverse phenolic composition and concentration. Food Res. Int..

[7] Huber K., B Brigide P., Bretas E.B., Canniatti-Brazaca S.G. (2016). Phenolic Acid, flavonoids and antioxidant activity of common brown beans (*Phaseolus vulgaris* L.) before and after cooking. J. Nutr. Food Sci..

[8] Akond G.M., Khandaker L., Berthold J., Gates L., Peters K., Delong H., Hossain K. (2011). Anthocyanin, total polyphenols and antioxidant activity of common bean. Am. J. Food Technol..

[9] Batista K.A., Prudencio S.H., Fernandes K.F. (2010). Changes in the functional properties and antinutritional factors of extruded hard-to-cook common beans (*Phaseolus vulgaris*, L.). J. Food Sci..

[10] De Mejia E.G., Guzmán-Maldonado S.H., Acosta-Gallegos J.A., Reynoso-Camacho R., Ramírez-Rodríguez E., Pons-Hernández J.L., González-Chavira M.M., Castellanos J.Z., Kelly J.D. (2003). Effect of cultivar and growing location on the trypsin inhibitors, tannins, and lectins of common beans (*Phaseolus vulgaris* L.) grown in the semiarid highlands of Mexico. J. Agric. Food Chem..

[11] McGuire R.G. (1992). Reporting of objective color measurements. HortScience.

[12] Hernández-López V.M., Vargas-Vázquez M.L.P., Muruaga-Martínez J.S., Hernández-Delgado S., Mayek-Pérez N. (2013). Origin, domestication and diversification of common beans, advances and perspectives. Rev. Fitotec. Mex..

[13] Singh S.P., Gepts P., Debouck D.G. (1991). Races of common bean (*Phaseolus vulgaris*, Fabaceae). Econ. Bot..

[14] Sinkovic L., Pipan B., Sinkovic E., Meglic V. (2019). Morphological seed characterization of common (*Phaseolus vulgaris* L.) and runner (*Phaseolus coccineus* L.) bean germplasm: A Slovenian gene bank example. BioMed Res. Int..

[15] Schmit V., Debouck D.G. (1991). Observations on the origin of *Phaseolus polyanthus* Greenman. Econ. Bot..

[16] Akillioglu H.G., Karakaya S. (2010). Changes in total phenol, total flavonoids, and antioxidant activities of common beans and pinto beans after soaking, cooking and *in vitro* digestion process. Food Sci. Biotechnol..

[17] Bernardino-Nicanor A., Acosta-García G., Güemes-Vera N., Montañez-Soto J.L., de Los Ángeles Vivar-Vera M., González-Cruz L. (2017). Fourier transform infrared and Raman spectroscopic study of the effect of the thermal treatment and extraction methods on the characteristics of Ayocote bean starches. J. Food Sci. Technol..

[18] Alvarado-López A.N., Gómez-Oliván L.M., Heredia J.B., Baeza-Jiménez R., García-Galindo H.S., Lopez-Martinez L.X. (2019). Nutritional and bioactive characteristics of Ayocote bean (*Phaseolus coccineus* L.): An underutilized legume harvested in Mexico. Cy TA J. Food.

[19] Holecek M., Vodenicarovova M. (2020). Effects of histidine supplementation on amino acid metabolism in rats. Physiol. Res..

[20] Xu B.J., Chang S.K.C. (2007). A comparative study on phenolic profiles and antioxidant activities of legumes as affected by extraction solvents. J. Food Sci..

[21] Capistrán-Carabarin A., Aquino-Bolaños E.N., García-Díaz Y.D., Chávez-Servia J.L., Vera-Guzmán A.M., Carrillo-Rodríguez J.C. (2019). Complementarity in Phenolic Compounds and the Antioxidant Activities of *Phaseolus coccineus* L. and *P. vulgaris* L. Landraces. Foods.

[22] Díaz A.M., Caldas G.V., Blair M.W. (2010). Concentrations of condensed tannins and anthocyanins in common bean seed coats. Food Res. Inter..

[23] Díaz-Batalla L., Widholm J.M., Fahey G.C., Castaño-Tostado E., Paredes-López O. (2006). Chemical components with health implications in wild and cultivated Mexican common bean seeds (*Phaseolus vulgaris* L.). J. Agric. Food Chem.

[24] Choung M.G., Choi B.R., An Y.N., Chu Y.H., Cho Y.S. (2003). Anthocyanin Profile of Korean cultivated kidney bean (*Phaseolus vulgaris* L.). J. Agric. Food Chem..

[25] Giusti F., Capuano E., Sagratini G., Pellegrini N. (2019). A comprehensive investigation of the behaviour of phenolic compounds in legumes during domestic cooking and *in vitro* digestion. Food Chem..

[26] Beninger C.W., Hosfield G.L. (2003). Antioxidant activity of extracts, condensed tannin fractions, and pure flavonoids from *Phaseolus vulgaris* L. seed coat color genotypes. J. Agric. Food Chem..

[27] Abdel-Aal E.S.M., Hucl P. (1999). A rapid method for quantifying total anthocyanins in blue aleurone and purple pericarp wheats. Cereal Chem..

[28] Tovar-Pérez E.G., Guerrero-Becerra L., Lugo-Cervantes E. (2017). Antioxidant activity of hydrolysates and peptide fractions of glutelin from cocoa (*Theobroma cacao* L.) seed. CyTA J. Food.

[29] Brand-Williams W., Cuvelier M.E., Berset C. (1995). Use of a free radical method to evaluate antioxidant activity. LWT.

[30] Samtiya M., Aluko R.E., Dhewa T. (2020). Plant food anti-nutritional factors and their reduction strategies: An overview. Food Prod. Process. Nutr..

[31] E Lacerda R.R., do Nascimiento E.S., de Lacerda J.T., Pinto L.D., Rizzi C., Bezerra M.M., Pinto I.R., Filho S.M., Pinto V.P., Filho G.C. (2017). Lectin from sedes of a Brazilian lima bean variety (*Phaseolus lunatus* L. var. cascabel) presentes antioxidant, antitumour and gastroprotective activities. Int. J. Biol. Macromol..

[32] Carbas B., Machado N., Oppolzer D., Ferreira L., Queiroz M., Brites C., Rosa E.A., Barros A.I. (2020). Nutrientes, Antinutrients, Phenolic Composition, and Antioxidant Activity of Common Bean Cultivars and their Potential for Food Applications. Antioxidants.

[33] Cruz-Bojórquez R.M., Coop-Gamas F.Y., Cárdenas-García S., Ávila-Escalante M.L. (2019). Perception of body image in Maya Adolescents and its Relationship with Body Dissatisfaction and nutritional status. J. Nutr. Food Sci..

[34] AOAC, Association of Analytical Communities (1995). Official Methods of Analysis of AOAC International.

[35] AOAC, Association of Analytical Communities (2006). Official Methods of Analysis Amino Acids Analysis.

[36] Broadhurst R.B., Jones W.T. (1978). Analysis of condensed tannins using acidified vanillin. J. Sci. Food Agric..

[37] Sun B., Ricardo-da-Silva J.M., Spranger I. (1998). Critical factors of vanillin assay for catechins and proanthocyanidins. J. Agric. Food Chem..

[38] De Mejia E.G., Hankins C.N., Paredes-Lopez O., Shannon L.M. (1990). The lectins and lectin-like proteins of tepary beans (*Phaseolus acutifolius*) and tepary-common bean (*Phaseolus vulgaris*) hybrids. J. Food Biochem..

[39] Kakade M.L., Rackis J.J., McGhee J.E., Puski G. (1974). Determination of trypsin inhibitor activity of soy products: A collaborative analysis of an improved procedure. Cereal Chem..

